# A dynamic prediction model for preeclampsia using the sFlt-1/PLGF ratio combined with multiple factors

**DOI:** 10.1186/s12884-024-06627-4

**Published:** 2024-06-26

**Authors:** Guili Chen, Yuanyuan Chen, Yao Shi, Zhoufen Mao, Jiaqi Lou, Jianting Ma

**Affiliations:** 1 The People’s Hospital of Yuyao, Zhejiang, 315400 China; 2 Health Science Center, Ningbo University, Zhejiang, 315000 China; 3 Ningbo University Medical Department, Zhejiang, 315000 China

**Keywords:** Preeclampsia(PE), SFlt-1/PLGF ratio, Dynamic prediction model, Logistic models

## Abstract

**Objective:**

Preeclampsia (PE) is a pregnancy-related multi-organ disease and a significant cause of incidence rate and mortality of pregnant women and newborns worldwide. Delivery remains the only available treatment for PE. This study aims to establish a dynamic prediction model for PE.

**Methods:**

A total of 737 patients who visited our hospital from January 2021 to June 2022 were identified according to the inclusion and exclusion criteria, forming the primary dataset. Additionally, 176 singleton pregnant women who visited our hospital from July 2022 to November 2022 comprised the verification set. We investigated different gestational weeks of sFlt-1/PLGF (soluble FMS-like tyrosine kinase-1, placental growth factor) ratio combined with maternal characteristics and routine prenatal laboratory results in order to predict PE in each trimester. Multivariate logistic regression was used to establish the prediction model for PE at different gestational weeks. The discrimination, calibration, and clinical validity were utilized to evaluate predictive models as well as models in external validation queues.

**Results:**

At 20–24 weeks, the obtained prediction model for PE yielded an area under the curve of 0.568 (95% confidence interval, 0.479–0.657). At 25–29 weeks, the obtained prediction model for PE yielded an area under the curve of 0.773 (95% confidence interval, 0.703–0.842)and 0.731 (95% confidence interval, 0.653–0.809) at 30–34 weeks. After adding maternal factors, uterine artery pulsation index(Ut-IP), and other laboratory indicators to the sFlt-1/PLGF ratio, the predicted performance of PE improved. It found that the AUC improved to 0.826(95% confidence interval, 0.748 ∼ 0.904) at 20–24 weeks, 0.879 (95% confidence interval, 0.823 ∼ 0.935) at 25–29 weeks, and 0.862(95% confidence interval, 0.799 ∼ 0.925) at 30–34 weeks.The calibration plot of the prediction model indicates good predictive accuracy between the predicted probability of PE and the observed probability. Furthermore, decision-curve analysis showed an excellent clinical application value of the models.

**Conclusion:**

Using the sFlt-1/PLGF ratio combined with multiple factors at 25–29 weeks can effectively predict PE, but the significance of re-examination in late pregnancy is not significant.

**Supplementary Information:**

The online version contains supplementary material available at 10.1186/s12884-024-06627-4.

## Introduction

PE is a pregnancy-related multi-organ disease and a significant cause of maternal and neonatal morbidity and mortality worldwide, especially in developing countries [1]. PE can be defined as new-onset hypertension after 20 weeks of gestation (systolic blood pressure persistently ≥ 140 mm Hg or diastolic blood pressure persistently ≥ 90 mm Hg, or both) associated with proteinuria, or end-organ dysfunction, or both. Pregnant women with pre-existing chronic hypertension will be diagnosed with chronic hypertension complicated by PE if they develop new-onset proteinuria or end-organ dysfunction [2]. Early PE is diagnosed if the disease occurs at ≤ 34 weeks of gestation [3]. The pathophysiology of PE is not fully understood. It may be a combination of genetic and environmental factors and abnormal placental formation [4]. Impaired spiral arteriole remodeling, maternal vascular malperfusion, and abnormal cellular interactions in the early first trimester might increase the risk of developing PE. As pregnancy enters the second trimester, the diseased placenta progressively gradually secretes large amounts of anti-angiogenic factors, such as soluble fms-like tyrosine kinase-1 (sFlt1) that cause vascular inflammation, endothelial dysfunction, and maternal vascular injury [2, 4–7]. Many studies have shown that circulating placental growth factor (PLGF) levels were decreased, and sFlt-1 levels were increased in PE. Predictions of PE were challenging because little is known about its etiology, various risk factors, and possible multiple pathogenic phenotypes. Previous studies have mostly focused on nested case-control studies [8], univariate analysis [9], or detection of the sFlt-1/PLGF ratio at a certain gestational week [10]. However, there have been relatively few predictions from continuous longitudinal and multivariate studies of sFlt-1/PLGF. A single detection indicator has limitations in predicting PE. Our study aims to investigate and compare the value of the sFlt-1/PLGF ratio at different gestational weeks combined with multiple factors in diagnosing PE, identify women at high risk of PE and provide early intervention to prevent and reduce adverse pregnancy outcomes.

## Materials and methods

This observational, single-center, prospective study was conducted at the People’s Hospital of Yuyao (Zhejiang, China). The study included single pregnant women who came to our hospital for examination between 11 and 13 weeks. Pregnant women with fetal abnormalities, fetal loss, and transfer to other hospitals for examination and delivery due to other reasons were excluded from the study.

This study enrolled single pregnant women who visited our hospital from January 2021 to June 2022. All participants underwent routine prenatal examinations and were followed up until delivery. A total of 737 patients met the inclusion and exclusion criteria and comprised the training sets. From July 2022 to November 2022, an independent validation set of 176 singleton pregnant women was screened using the same criteria as the development dataset. All eligible women attending our hospital visit at 11 to 13 weeks gestation were provided with written information about the study, and those who agreed to participate provided written informed consent.

The maternal characteristics of pregnant women were obtained through consultation with professional medical practitioners during the initial visit to our hospital. This included maternal age, pre-pregnancy body mass index (BMI), mode of pregnancy, number of pregnancies, mean arterial pressure (MAP), previous delivery history, abortion history, previous pregnancy with gestational hypertension, history of chronic hypertension, chronic kidney disease, and immune system diseases. We also assessed whether aspirin was being taken during this pregnancy and checked for gestational diabetes and hypothyroidism due to pregnancy comorbidity. At 11 to 13 weeks gestation, laboratory indicators including pregnancy associated plasma protein-A (PAPP-A), PAPP-A MOM, hematocrit (HCT), fasting blood glucose (FBG), hemoglobin (Hb), and 25-hydroxyvitamins D were measured. Doppler ultrasound examinations were performed transabdominally to measure the pulsatility index and calculate the mean UtA-PI of the left and right arteries. Between 20 and 24 weeks of pregnancy, we checked for triglycerides(TG), total cholesterol(TC), uric acid(UC), and sFlt-1/PLGF. Then we re-measured sFlt-1/PLGF every five weeks at pregnancy 25–29 weeks and again at pregnancy 30–34 weeks.

All data were analyzed using SPSS 26.0 and R 4.2.2 software. The normality of the variables was assessed using either the Kolmogorov-Smirnov or Shapiro-Wilk test. Descriptive statistics were used to characterize the participants’ essential features. Group comparisons based on variable normality were conducted using Independent t-tests or Mann-Whitney U-tests. Categorical variables were summarized by counts and percentages, and compared using either Pearson’s chi-square or Fisher’s exact test. Potential risk factors for PE were identified as the best predictors through binary Logistic regression analysis, leading to the establishment of a logistic prediction model. The predictive performance was evaluated using receiver operating characteristic (ROC) curves and calibration plots, which assessed the consistency between predicted PE probabilities and actual outcomes. External validation of the model was performed with populations from different time periods within the same region, in addition to decision-curve analysis (DCA) being utilized to evaluate clinical validity. A probability value less than 0.05 was considered statistically significant.

## Result

A total of 737 pregnant women with singleton pregnancies were included in the study, of which 55 (7.5%) ultimately developed PE and were assigned to the study group. Among these, 3 pregnant women (0.41%) had early-onset PE, while the remaining 52 had late-onset PE. In the external test population of 176 cases, 13 patients (7.4%) had PE, with one pregnant woman (0.56%) having early-onset PE and the remaining 12 having late-onset PE. The basic sociodemographic characteristics, medical history, and laboratory data of all participants are presented in Table 1. The age and nulliparous proportion of the PE patients were higher than those in the normal group (*p* < 0.05). However, levels of PAPP-A and PAPP-A MOM were significantly lower in the PE patients (*p* < 0.05). Pre-pregnancy BMI, MAP, hematocrit, fasting blood glucose, triglyceride, and uric acid levels were significantly higher in the PE patients compared to the normal group. The UtA-PI of the PE group was also significantly higher than that of the control group. Significant differences between the two groups were observed in aspirin use, gestational diabetes incidence, history of original hypertension, and mode of pregnancy(*p* < 0.05).

In Table 1, it is evident that the sFlt-1/PLGF ratio varies across different gestational weeks. Specifically, the sFlt-1/PLGF ratio in the PE group was higher than that of the control group at 20–24 weeks, although this difference was not statistically significant (*p* = 0.096). However, at 25–29 weeks and 30–34 weeks of pregnancy, the sFlt-1/PLGF ratio in the PE group was significantly higher than that of the control group (*p* < 0.001).

**Table 1:** Sociodemographic characteristics of the PE group and the control group

Characteristics	Control(*n* = 682)	PE(*n* = 55)	*P* value
**Age(year)**	27(23 ∼ 30)	27(25 ∼ 31)	0.004
**Pre-pregnancy BMI(kg/m** ^**2**^ **)**	21.34(19.52 ∼ 23.44)	22.58(20.7 ∼ 27.33)	0.002
**MAP (mmHg)**	82.83(76.67 ∼ 88.33)	85.33(80 ∼ 93.5)	0.000
**FBG(mmol/L)**	4.23(4.02 ∼ 4.43)	4.42(4.25 ∼ 4.77)	0.000
**TG(mmol/L)**	2.24(1.8 ∼ 2.94)	3.09(2.34 ∼ 3.94)	0.000
**TC(mmol/L)**	5.68(5.02 ∼ 6.28)	5.19(4.78 ∼ 6.25)	0.761
**Uric acid(µmol/L)**	256(224 ∼ 293)	296(239 ∼ 368)	0.000
**Hb(g/L)**	114(109 ∼ 120)	115(105.5 ∼ 123)	0.231
**Albumin(g/L)**	37.2(35.9 ∼ 38.6)	37(35.35 ∼ 38.4)	0.075
**25 hydroxyvitamins D**	41.19(31.5 ∼ 52.9)	36.45(26.64 ∼ 50.63)	0.856
**PAPPA**	3860(2773 ∼ 5885)	2420(1810 ∼ 5300)	0.020
**PAPPA(MoM)**	1.06(0.72 ∼ 1.43)	0.67(0.51 ∼ 1.05)	0.000
**Hct(%)**	37.1(34.9 ∼ 38.98)	38.7(36.9 ∼ 40.35)	0.000
**UlA PI**	1.69(1.49 ∼ 1.95)	1.96(1.57 ∼ 2.56)	0.001
**sFlt-1/PLGF ratio**			
**20 **∼** 24 W**	22.12(17.27 ∼ 27.57)	25.82(18.92 ∼ 32.83)	0.096
**25 **∼** 29 W**	17.05(10.36 ∼ 24.88)	28.74(24.3 ∼ 38.61)	0.000
**30 **∼** 34 W**	19.39(12.01 ∼ 26.34)	35.67(21.29 ∼ 38.7)	0.000
**Medical history, n(%)**			
**Nulliparous, n(%)**			0.001
**Yes**	282(41.35)	36(65.45)	
**No**	400(58.65)	19(34.55)	
**Using aspirin, n(%)**			0.000
**Yes**	61(8.9)	13(23.6)	
**No**	621(91.1)	42(76.4)	
**Gestational diabetes mellitus, n(%)**			0.000
**Yes**	43(6.3)	18(32.7)	
**No**	639(93.7)	37(67.3)	
**Chronic hypertension, n(%)**			0.000
**Yes**	2(0.3)	10(18.2)	
**No**	680(99.7)	45(81.8)	
**Gestational hypertension, n(%)**			0.322
**Yes**	4(0.6)	1 (1.8)	
**No**	678(99.4)	54(98.2)	
**chronic renal disease, n(%)**			0.322
**Yes**	4(0.6)	1 (1.8)	
**No**	678(99.4)	54(98.2)	
**Pregnancy mode, n(%)**			0.000
**nature conceived**	667 (97.8)	48(87.3)	
**Assisted reproductive treatment**	15(2.2)	7(12.7)	
**Autoimmune Disease, n(%)**			0.068
**Yes**	4(0.6)	2 (3.6)	
**No**	678(99.4)	53(96.4)	
**hypothyroidism, n(%)**			0.190
**Yes**	43(6.3)	6 (10.9)	
**No**	639(93.7)	49(89.1)	
**abortion history, n(%)**			0.269
**Yes**	338(49.6)	23(41.8)	
**No**	344(50.4)	32(58.2)	

Analyzed by using the Mann-Whitney U test

sFlt-1: pg/ml; PLGF: pg/ml

The results of the single-factor logistic analysis using the collected data are presented in Table 2. In addition to the sFlt-1/PLGF ratio, significant factors between the two groups included age, PAPP-A MOM, HCT, pre-pregnancy BMI, FBG, UC, UtA-PI, aspirin use, diabetes in pregnancy, history of delivery, primary hypertension and mode of pregnancy. Variables significantly associated with PE in univariate logistic regression were included in multivariate logistic regression. Six independent predictive factors were identified (Table 3), including sFlt-1/PLGF ratio at 25–29 weeks and 30–34 weeks, UtA-PI, PAPP-A MOM, uric acid levels, history of delivery and primary hypertension. The collinearity test results indicated that all variables had variance inflation factors less than 2, which suggests that collinearity can be ignored.

**Table 2:** Univariable analysis of the PE and the normal groups

Variable	*P* value	OR (95% CI)
**sFlt-1/PLGF ratio**		
**20 **∼** 24w**	0.314	1.016(0.985 ∼ 1.048)
**25 **∼** 29w**	0.000	1.093(1.065 ∼ 1.122)
**30 **∼** 34w**	0.000	1.080(1.053 ∼ 1.108)
**Age(year)**	0.002	1.076(1.026 ∼ 1.129)
**Pre-pregnancy BMI(kg/m** ^**2**^ **)**	0.001	1.104(1.042 ∼ 1.170)
**MAP(mmHg)**	0.096	1.009(0.998 ∼ 1.019)
**FBG(mmol/L)**	0.000	3.555(2.092 ∼ 6.041)
**TG(mmol/L)**	0.976	1(0.982 ∼ 1.018)
**Uric acid(µmol/L)**	0.000	1.010(1.006 ∼ 1.015)
**PAPPA**	0.320	1(1 ∼ 1)
**PAPPA(MoM)**	0.023	0.447(0.224 ∼ 0.894)
**Hct(%)**	0.000	1.233(1.117 ∼ 1.362)
**UlA PI**	0.000	4.451(2.458–8.060)
**Nulliparous, n(%)**	0.001	2.688(1.510 ∼ 4.782)
**Using aspirin, n(%)**	0.001	3.151(1.604 ∼ 6.191)
**Gestational diabetes mellitus, n(%)**	0.000	7.229(3.803 ∼ 13.742)
**Chronic hypertension, n(%)**	0.000	75.556(16.071 ∼ 355.219)
**mode of Pregnancy, n(%)**	0.000	6.485(2.524 ∼ 16.663)

**Table 3:** Multivariable logistic regression analysis based on the data

Variable	β-coefficient	*P* value	OR (95% CI)
**sFlt-1/PLGF ratio**			
**20 **∼** 24w**	0.032	0.168	1.033(0.986 ∼ 1.082)
**25 **∼** 29w**	0.082	0.000	1.085(1.05 ∼ 1.122)
**30 **∼** 34w**	0.082	0.000	1.086(1.047 ∼ 1.126)
**PAPP-A MOM**	-0.957	0.023	0.384(0.168 ∼ 0.876)
**Uric acid**	0.011	0.001	1.011(1.004 ∼ 1.017)
**UtA-PI**	1.493	0.000	4.451(1.961 ∼ 10.099)
**Nulliparous**	-1.355	0.002	0.258(0.109 ∼ 0.609)
**chronic hypertension**	3.661	0.002	38.915(4.004 ∼ 378.246)

**Fig. 1:**
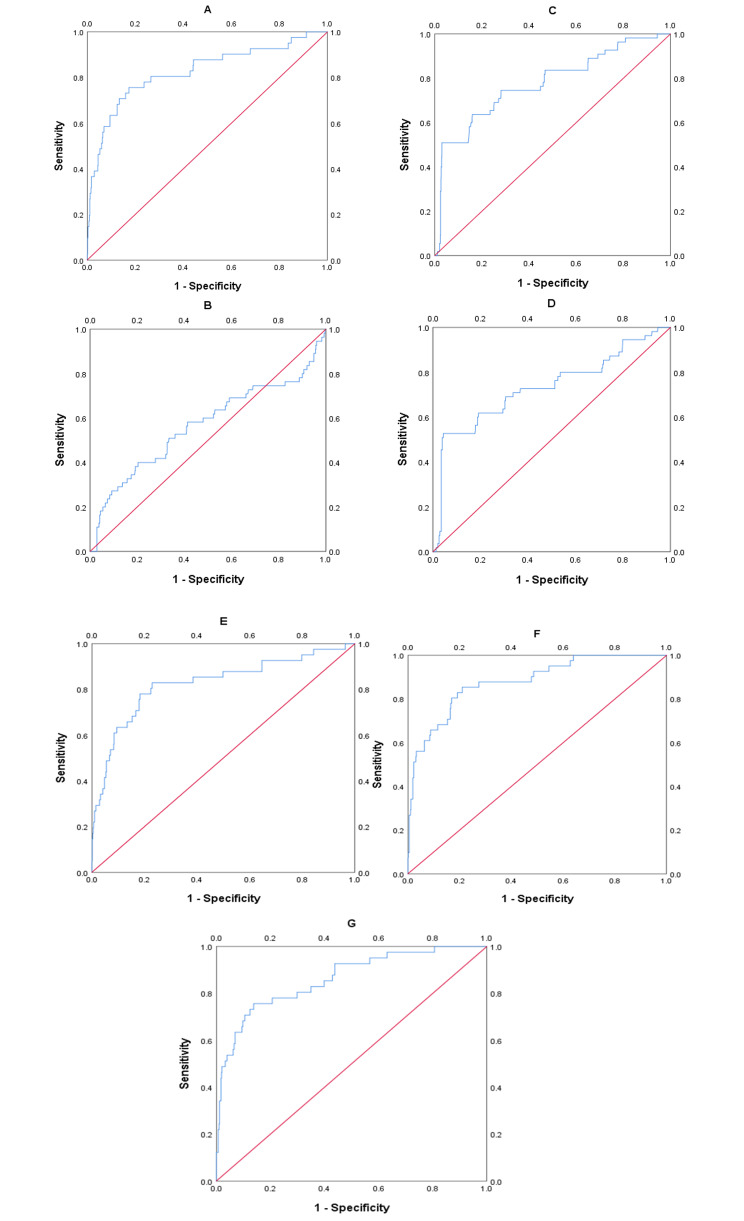
ROC curves of maternal factors, single-factor and multi-factor validation models. **A** shows the ROC curve of the maternal factors and UtA-PI. **B** shows the ROC curve of the predictive model for the sFlt-1/PLGF ratio from 20 to 24 weeks, **C** from 25 to 29 weeks, and **D** from 30 to 34 weeks. Figure **E, F, G** shows the ROC curve of the multi-factor prediction model corresponding to gestational age

**Fig. 2:**
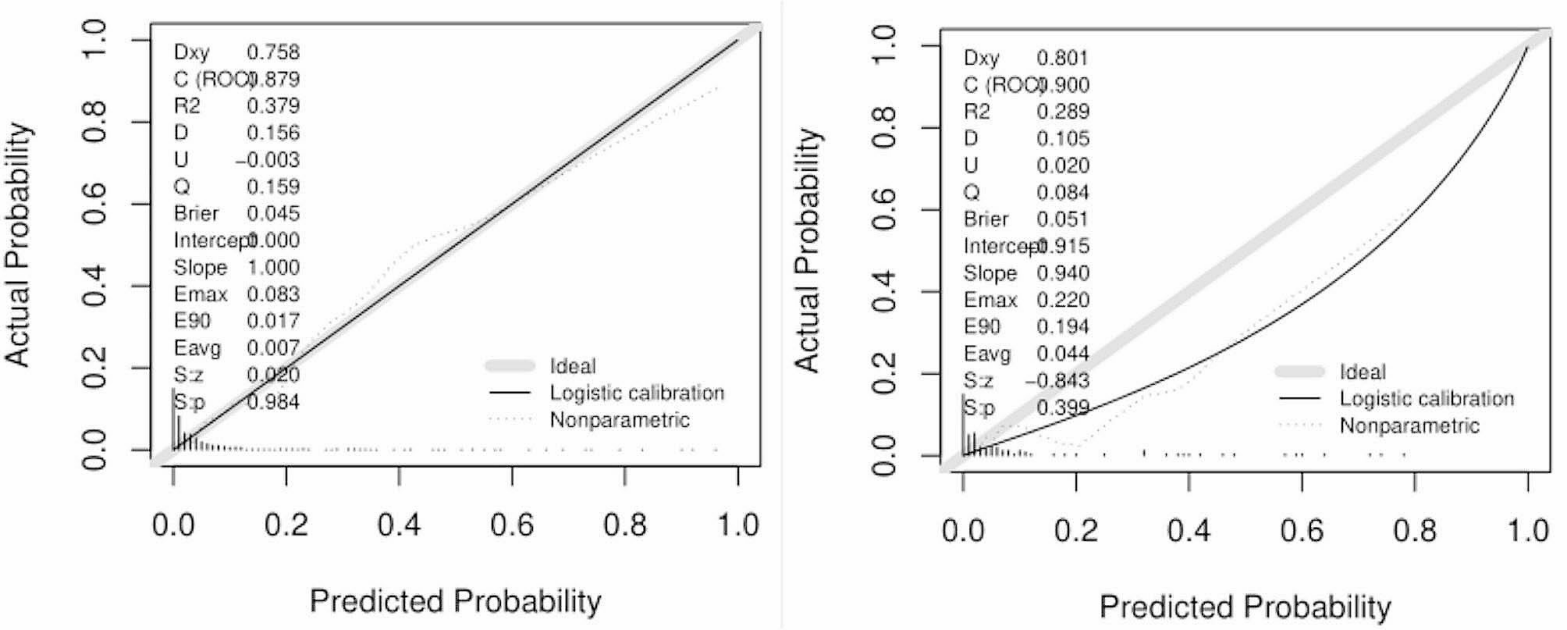
Calibration curves. Validation models for all predictive factors. The left image shows the calibration map of the prediction model, and the right image shows the external validation calibration map

**Fig. 3:**
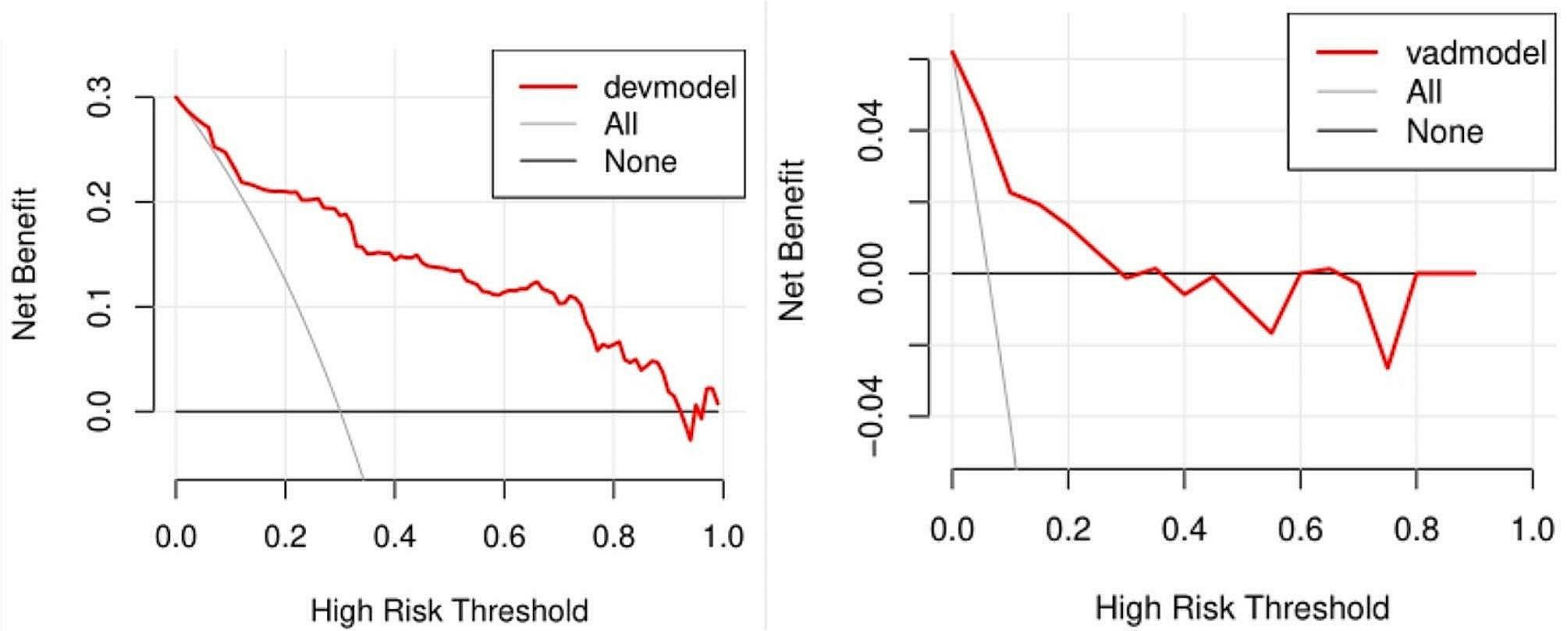
Decision curve analysis. On the left is the DCA of the prediction model, and on the right is the DCA of external validation

The area under the ROC curve (AUC) of the predictive model for PE obtained at 11 ∼ 13 weeks of pregnancy with maternal factors was 0.815 (95% confidence interval, 0.731 ∼ 0.898), with a sensitivity of 75.6% and a false positive rate of 18.9% (Fig. 1A; Table 4). The AUC for sFlt-1/PLGF in predicting PE at 20 ∼ 24 weeks gestation was closer to 0.5 (Fig. 1B; Table 4), indicating poor sensitivity and specificity. At 25–29 weeks of pregnancy, the AUC of the predictive model for PE with sFlt-1/PLGF ratio was found to be 0.776 (95% confidence interval, 0.704–0.848), with a sensitivity of 50.9% and a false positive rate of only 3.1%. The cut-off value for sFlt-1/PLGF is set at 34(Fig 1C, Table 4). The AUC of the predictive model for PE obtained at 30–34 weeks of pregnancy with sFlt-1/PLGF ratio was 0.732(95% confidence interval, 0.65–0.814), with a sensitivity of 52.7% and a false positive rate of 4.4%. The cut-off value of sFlt-1/PLGF is 34(Fig. 1D; Table 4). We combined other predictors to sFlt-1/PlGF using multiple logistic regression analysis to compare its performance. It found that the AUC improved to 0.826(95% confidence interval, 0.748 ∼ 0.904) at 20–24 weeks(Fig. 1E; Table 4), 0.879 (95% confidence interval, 0.823 ∼ 0.935) at 25–29 weeks(Fig. 1F; Table 4), and 0.862(95% confidence interval,0.799 ∼ 0.925) at 30–34 weeks(Fig. 1G; Table 4). The calibration curve of the model (Fig. 2, left) shows a calibration slope of 1 and an intercept of 0, indicating a good prediction accuracy between the predicted probability of PE and the observed probability. However, the calibration plot of the external validation model (Fig. 2, right) shows a calibration slope of 0.940 and an intercept of -0.915, indicating the sensitivity and accuracy are relatively poor. The DCA plot indicated good positive net benefits of the predictive model (Fig. 3, left) and External validation model (Fig. 3, right) among majority threshold probabilities.

**Table 4:** Ability of the single-factor and multi-factor to predict PE analysis based on roc curves

Variable	AUC	*P*	Cut-off	OR(95%CI)	Sensitivity	1-Specificity	Yoden index
**maternal factors + Ut-PI**							
**11–13 W**	0.815	0	0.077	(0.731 ∼ 0.898)	75.60%	18.90%	0.567
**sFlt/PIGF**							
**20–24 W**	0.567	0.096	28	(0.474 ∼ 0.661)	40.0%	20.40%	0.196
**25–29 W**	0.776	0	34	(0.704 ∼ 0.848)	50.9%	3.10%	0.478
**30–34 W**	0.732	0	34	(0.65 ∼ 0.814)	52.7%	4.40%	0.483
**sFlt/PIGF + maternal factors + Ut-PI**							
**20–24 W**	0.826	0	0.067	(0.748 ∼ 0.904)	82.90%	22.90%	0.6
**25–29 W**	0.879	0	0.057	(0.823 ∼ 0.935)	85.40%	21.00%	0.644
**30–34 W**	0.862	0	0.088	(0.799 ∼ 0.925)	75.60%	13.80%	0.618

## Discuss

PE occurs in 2–8% of pregnancies worldwide, with estimates of at least 16% among low- and middle-income countries [5, 10]. Our study’s findings indicate an incidence rate of PE at 7.5%, which is consistent with previous literature reports. The impact of PE on the health of mothers and newborns is severe. Women whoexperience PE during pregnancy appear tohavesignificantly increased future risk of vascular dementia [11]. Maternal hypertensive disorders of pregnancy(HDP), other than gestational hypertension, independently increase the risk of intellectual disability in offspring, in addition to the potential impacts of preterm birth and small-for-gestational-age (SGA) [12]. Currently, delivery remains the only available treatment for PE. Therefore, it is particularly important to predict the occurrence of PE early and provide preventive measures.

Many studies have found that angiogenic biomarker tests conducted in early pregnancy or before 20 weeks of gestation do not effectively predict the onset of PE [3, 5, 8, 9]. Ohkuchi et al. [13] discovered that serum PlGF levels in women with singleton pregnancy at 9–13 weeks of gestation may be useful for predicting preterm PE. Additionally, Amylidi-Mohr’s study [14] demonstrated the effectiveness of first-trimester combined screening for PE using the FMF algorithm in their population. Guizani et al. [15] utilized the Fetal Medicine Foundation (FMF) at 11–13 weeks gestation to predict PE and found a detection rate of 80.6% for PE occurring before 37 weeks and 31.8% for PE occurring after 37 weeks when screening by maternal factors, MAP, UtA-PI, and sFlt. In our study, a combination of maternal factors and UtA-PI showed good predictive performance for precursors between 11 and 13 weeks (0.815;95% confidence interval:0.731–0.898). Several studies [16, 17] have also shown that high-risk women who begin taking daily aspirin in early pregnancy significantly reduce their risk of developing PE, particularly premature PE. In our study, pregnant patients with high-risk factors for PE and those predicted to have PE at 11–13 weeks were administered a daily dose of 75 mg aspirin before 16 weeks, which was continued until 36 weeks of pregnancy. This prophylactic treatment resulted in a reduced incidence rate of early-onset PE (0.54%). According to Yemane’s study, the incidence of GH was 6% (4.9–8.5), and the likelihood of progression from GH to PE was 17.1% (13.4–23.8). Our data showed no statistically significant difference in previous GH history between the two groups, which may be attributed to the administration of aspirin prophylactic treatment to pregnant women with high-risk factors.

Some research studies [15, 18, 19] have indicated that the FMF algorithm is effective in predicting preterm PE. However, its performance in predicting late-onset PE ≥ 37 weeks is found to be poor. Nevertheless, both early-onset and late-onset PE lead to severe short-term and long-term complications for pregnant women and newborns. Therefore, we included TG, TC, and UC, as well as sFlt-1/PLGF levels detected during pregnancy at 20–24 weeks. Additionally, we repeated the measurement of sFlt-1/PLGF levels during pregnancy at 25–29 weeks and again at 30–34 weeks.

In our study, the levels of sFlt-1/PLGF in the PE group increased with gestational age and were significantly higher than those in the control group at 24–29 weeks and 30–34 weeks of pregnancy. Levine et al. [20] found that the sFlt1/PLGF ratio was significantly higher at 25 through 28 weeks among those who developed term PE compared to the controls. Moore Simas et al. [21] conducted a prospective study on 94 pregnant women between 22 and 36 weeks, and they observed that, compared to the control group, the increase in sFlt1/PLGF ratio in PE women became more significant with increasing gestational age. These findings are consistent with our research results. Fan Yu et al. [9] tested sFlt-1 and PLGF in pregnant women in southwestern China between 12 and 36 weeks of pregnancy (an average of 29 weeks) and used their ratio for predicting PE. They discovered that when the critical value of sFlt-1/PLGF ratio was set at 26.6, the area under the ROC curve was calculated as being high at 0.918, with both high sensitivity (85.42%) and specificity (96.27%). Karoline et al. [22] reported that the cut-off value for predicting PE in Austrians using sFlt-1/PLGF at 32 weeks of pregnancy was 10.3. In a cohort study of 97 pregnant women at gestational ages of 24–28 weeks in the south of Vietnam, Trung et al. [10] found that the sFlt-1/PLGF ratio had an area under the ROC curve of 80%, modest sensitivity (50.0%), and good specificity (86.6%) with a cut-off point of 4.3. Additionally, Viktorija et al. [23] conducted a case-control clinical study at the tertiary care center of the Hospital of Lithuanian University of Health Sciences Kauno Klinikos. On the day of PE diagnosis, the SFlt-1/PLGF ratio was tested and found to have a cut-off value of ≥ 35 (sensitivity 95.8%, specificity 96.2%) when the sensitivity and specificity for predicting PE were highest. Villa [24] conducted a nested case-control study on high-risk women and found that Serum sFlt-1/PlGF ratio over 40 at 26 + 0 to 28 + 0 weeks of gestation has high specificity and sensitivity in identifying women who developed early-onset PE disease. These studies demonstrate that cut-off values for diagnosis vary among different countries and regions, possibly due to factors such as race, genetics, living environments, and habits which are speculated to be related to the pathogenesis of PE. Our study aimed to investigate the dynamic changes in the sFlt-1/PLGF ratio during pregnancy from 20 to 34 weeks and its relationship with PE. The data revealed that in normal pregnant women, the sFlt-1/PLGF ratio peaked at 20 to 24 weeks of gestation, decreased until 25 to 29 weeks, and then gradually increased at 30 to 34 weeks. Conversely, in patients with PE, there was a significant increase in the sFlt-1/PLGF ratio as gestational age advanced from 20 to 34 weeks.

Dawson LM et al. [25] discovered that factors independently associated with an increased risk of PE included primiparous delivery, which aligns with our research findings. Furthermore, the SOGC clinical practice guidelines [26] also identify primiparous women as moderate risk factors. Our present results are consistent with several previous findings indicating that the Uric acid levels [27, 28] in patients with PE were higher than those in normal pregnant women. However, maternal serum PAPP-A MOM [29, 30] was significantly lower. Some studies [31–33] have demonstrated that the uterine artery pulsatility index measured by Doppler ultrasound is useful and effective for predicting PE and should be implemented in clinical practice. Chronic hypertension [34, 35] was associated with an increased risk of PE, which is in line with our research findings.

After incorporating maternal factors, angiogenic markers, and Ut-PI into the predictive factors at 25–29 weeks of gestation, the AUC for predicting PE increased to 0.879, with a sensitivity of 85.4% and a false positive rate of 21%. The AUC also increased to 0.862 at 30–34 weeks, with a sensitivity of 75.6% and a false positive rate of 13.8%. The use of multiple factors for predicting PE at 25–29 weeks demonstrated good performance; however, its performance was relatively poor at 30–34 weeks. Additionally, early-onset PE occurring between 30 and 34 weeks can be diagnosed without prediction. Our research suggests that combining the sFlt-1/PLGF ratio with multiple factors at 25–29 weeks effectively predicts PE, but re-examination in late pregnancy does not hold significant significance according to our findings. Our prediction model performed better in calibration and discrimination than another model(Maric, I. 2020) [36]. We made a DCA to quantify the clinical usefulness of the models and found that our prediction model exhibited good performance. However, the sensitivity and accuracy of the calibration map for external validation models are relatively poor. This may be due to the limited sample size in external validation model research.

The strength of our study lies in its integration of maternal factors, biochemical markers, and dynamic angiogenesis factors at 20–34 weeks for the prediction of PE. At 20–24 weeks, maternal factors and biochemical markers can compensate for the relatively weak predictive performance of the sFlt-1/PLGF ratio. However, it is important to note that one limitation of this study is the insufficient number of samples. Additionally, as a single-center study, we did not monitor the sFlt-1/PLGF ratio throughout pregnancy or assess biochemical markers at different gestational weeks.

## Conclusion

The sFlt-1/PLGF ratio combined with multiple factors at 25–29 weeks can effectively predict PE. However, the significance of re-examination in late pregnancy is not substantial.

### Electronic supplementary material

Below is the link to the electronic supplementary material.

Supplementary Material 1

Supplementary Material 2

## Data Availability

The datasets generated and/or analyzed during the current study are available from the corresponding author on reasonable request.

## Declarations

### Supplementary Information

PE Manuscript Supplementary Material 1.

PE Manuscript Supplementary Material 2.
